# Experimental Considerations for Single-Cell RNA Sequencing Approaches

**DOI:** 10.3389/fcell.2018.00108

**Published:** 2018-09-04

**Authors:** Quy H. Nguyen, Nicholas Pervolarakis, Kevin Nee, Kai Kessenbrock

**Affiliations:** ^1^Department of Biological Chemistry, University of California, Irvine, Irvine, CA, United States; ^2^Center for Complex Biological Systems, University of California, Irvine, Irvine, CA, United States

**Keywords:** single-cell genomics, single-cell analysis, cell isolation, computational biology, cellular heterogeneity

## Abstract

Single-cell transcriptomic technologies have emerged as powerful tools to explore cellular heterogeneity at the resolution of individual cells. Previous scientific knowledge in cell biology is largely limited to data generated by bulk profiling methods, which only provide averaged read-outs that generally mask cellular heterogeneity. This averaged approach is particularly problematic when the biological effect of interest is limited to only a subpopulation of cells such as stem/progenitor cells within a given tissue, or immune cell subsets infiltrating a tumor. Great advances in single-cell RNA sequencing (scRNAseq) enabled scientists to overcome this limitation and allow for in depth interrogation of previously unexplored rare cell types. Due to the high sensitivity of scRNAseq, adequate attention must be put into experimental setup and execution. Careful handling and processing of cells for scRNAseq is critical to preserve the native expression profile that will ensure meaningful analysis and conclusions. Here, we delineate the individual steps of a typical single-cell analysis workflow from tissue procurement, cell preparation, to platform selection and data analysis, and we discuss critical challenges in each of these steps, which will serve as a helpful guide to navigate the complex field of single-cell sequencing.

## Introduction

Elucidating cellular heterogeneity represents a major scientific challenge in many areas of biology and biomedical research including developmental and stem cell biology, immunology, neurobiology, and cancer research (Wagner et al., 2016). Recent convergence of next generation sequencing (NGS) and bioengineering approaches to manipulate individual cells has led to unbiased single-cell DNA (Navin et al., 2011), RNA (Pollen et al., 2014; Treutlein et al., 2014; Tanay and Regev, 2017), and ATAC (Buenrostro et al., 2015) sequencing. These technological advances are redefining our understanding of how biological systems function and have formed the basis for large-scale, international collaborations such as the Human Cell Atlas project (Rozenblatt-Rosen et al., 2017). In this spirit, a recent endeavor using microwell-based single-cell RNAseq (scRNAseq) created the first cell atlas to map out most tissues of the mouse (Han et al., 2018). Moreover, scRNAseq has provided critical new insights into key developmental processes such as the earliest steps of cardiovascular lineage segregation in mice (Lescroart et al., 2018), and our recent work utilized scRNAseq to reveal the spectrum of cellular heterogeneity within the human breast epithelium identifying three major cell types each harboring multiple distinct cell states (Nguyen et al., 2018).

**FIGURE 1:**
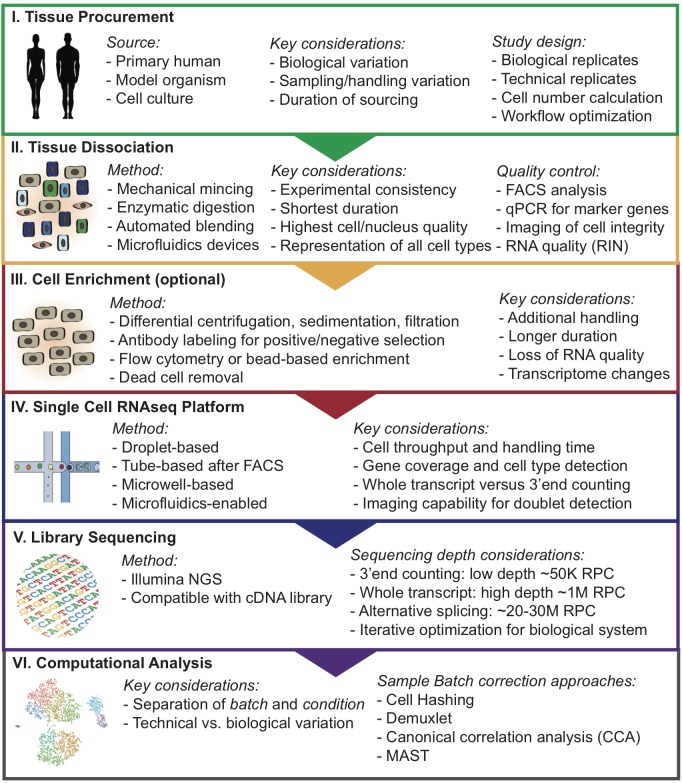
Overview of step-wise approach to designing single-cell analysis workflows. RNA integrity number (RIN); Reads per cell (RPC).

Due to the high sensitivity of these methods, in particular scRNAseq, it can be difficult to choose an adequate approach to minimize batch effects and unwanted technical variation that may overshadow true biological insights. Here, we provide helpful insights and delineate a step-wise approach for designing single-cell analysis workflows (**Figure 1**).

## Cell Dissociation and Single-Cell Preparation

The process of single-cell preparation is arguably the greatest source of unwanted technical variation and batch effects in any single-cell study (Tung et al., 2017). Different tissues can vary significantly in extracellular matrix (ECM) composition, cellularity, and stiffness, and therefore dissociation protocols must be optimized for the specific tissue type of interest. Conventional protocols for single-cell preparation typically involve the following steps: (1) tissue dissection, (2) mechanical mincing, (3) enzymatic/proteolytic ECM breakdown (e.g., dispase, collagenase, trypsin) often accompanied by mechanical agitation, and (4) optional enrichment for cell types of interest by flow cytometry, bead-based immune-selection, differential centrifugation, or sedimentation. Each step can affect the cells’ expression signatures, and should therefore be carefully optimized to introduce the least artifact. An optimal tissue dissociation protocol will yield as many viable cells as possible in the shortest possible duration without preferentially depleting or significantly altering the frequencies of certain cell types.

Recent advances in bioengineering of innovative microfluidic cell dissociation devices (Qiu et al., 2014) have the potential to radically change the way tissue samples are dissociated into single cells, while avoiding inter-assay variation due to human handling of the tissue. Several microfluidic devices have been optimized for streamlined tissue digestion, cell dissociation, filtering, and polishing. In brief, these devices were designed to work with tissue sequentially through progressively smaller size scales, starting from tissue specimen, through cellular aggregates and clusters, and finally eluting a solution containing close to 100% single cells, which will be ideal for scRNAseq applications. In addition, new semi-automated commercially available systems can help streamline tissue dissociation (e.g., Miltenyi gentleMACS). These devices offer tissue-type specific kits that may allow more reproducible, time-saving and efficient tissue dissociation and single-cell preparation (Meeson et al., 2013; Baldan et al., 2015). Ultimately, determining a “best practices” dissociation strategy through heuristic optimization will be critical for downstream single-cell library quality.

### Cell Type Enrichment

There are various methods for isolating specific cell populations or removal of unwanted populations that should be optimized for any specific tissues type. Manual isolation utilizing magnetic beads or gradient purification are potential methods for removal of unwanted cells such as dead cells. Flow cytometry is a widely used, high-throughput method to enrich for rare cells such as hematopoietic stem cells (Radbruch and Recktenwald, 1995; Will and Steidl, 2010). However, these methods are not without drawbacks, since they can introduce artificial stress on cells and change their expression profile (Van Den Brink et al., 2017). Methods that involve antibody binding for purification can also affect the cell expression profile if binding of the antibodies to cell surface molecules induce intracellular signaling (Kornbluth and Hoover, 1989; Christaki et al., 2011). Flow cytometry-isolated cells are exposed to high pressure during sorting and these osmotic and pressure changes introduced to cells during cell sorting and handling can induce change to the cell expression profile of multiple cell types (Xiong et al., 2002; Romero-santacreu et al., 2009; Van Den Brink et al., 2017).

### Quality Control

Due to the high cost of single-cell sequencing experiments, careful quality control measurements should be executed. The performance of alternative protocols can be assessed using a number of readouts. A useful first metric can be acquired using imaging of viability such as using the Countess platform (Thermo Fisher Scientific). Flow cytometry is particularly valuable to measure several critical metrics simultaneously, such as cell viability, and contamination with doublets and small cell clusters which can confound single-cell sequencing results. Flow cytometry can also be used to evaluate whether cell populations of interest, such as immune cells, stromal fibroblasts, or stem cell populations, are maintained in the cell preparation and in the appropriate frequency. Finally, an additional metric on RNA quality can be acquired using the RNA integrity number (RIN) method (Schroeder et al., 2006).

## Single-Cell Transcriptomic Platform

Protocols for transcriptome analysis have advanced rapidly, resulting in several robust methods which range in cell and mRNA capture strategy, barcoding, throughput, and level of automation (Fan et al., 2015; Macosko et al., 2015). Selection of the optimal approach depends largely on the research question. Recent high-throughput protocols for scRNAseq have dramatically increased scalability through automation, increasing the number of cells that can be processed simultaneously, and decreasing reagent cost through reaction miniaturization. Using microwell-based (Cytoseq, Wayfergen), microfluidics-based (Fluidigm C1 HT), or droplet-based (inDrop, Drop-seq, and 10× Chromium) approaches, hundreds to thousands of cells can be captured in a single experiment (Islam et al., 2014; Picelli et al., 2014; Klein et al., 2015; Heath et al., 2016; Zheng et al., 2017). The newest of these protocols utilize beads functionalized with oligonucleotide primers, which each contain a universal PCR priming site, a cell-specific barcode, an mRNA capture sequence, and Unique Molecular Identifiers (UMI). Individual cells are captured in wells or droplets with a single bead. Cell-specific barcode are similar within a droplet but unique UMI sequence on the primer allows for individual transcripts within a cell to be counted. This provides a quantitative readout of the number of transcripts of each gene detected in a cell, thereby reducing the effects of amplification duplicates that occur with earlier technologies (Ramsköld et al., 2012; Patel et al., 2014). High-throughput 3′-end counting approaches have several important limitations. Since only the 3′-end of each mRNA are sequenced, differential splicing analyses are not feasible (Macosko et al., 2015; Heath et al., 2016). High-throughput approaches typically only achieve ∼10% transcriptome coverage, relative to ∼40% for full-length scRNAseq protocols that use Switching Mechanism at 5′End of RNA Template (SMART) chemistry (Tirosh et al., 2016; Yuan et al., 2017). This is partly due to lower mRNA capture efficiency, but also due to lower sequencing depth. Single-cell qPCR platforms (e.g., Fluidigm C1 and Biomark) remain superior in sensitivity for detecting low-expressed genes (Lawson et al., 2015).

Protocols for processing rare cells usually involve an upstream capture step by flow cytometry or micromanipulation, followed by dispensing single cells into microtubes or microwell plates. Studies investigating rare cell populations that require selection via specific markers (e.g., adult tissue stem cell populations), are best performed using these protocols. Single-cell libraries are prepared using SMART-based chemistry, which utilizes a template-switching oligonucleotide (TSO) (Tirosh et al., 2016). This TSO can be used to prime off of the untemplated nucleotides added by the reverse transcriptase, enabling subsequent PCR using a single primer and capture of full length transcripts (Tirosh et al., 2016; Yuan et al., 2017). cDNAs are then amplified by PCR and libraries are prepared for sequencing using standard protocols. Although there have been several large scale projects utilizing these protocols, because they are manual in nature and utilize larger microliter reaction volumes, they limit the number of cells that can be processed at reasonable cost.

Another area of ongoing debate is how to determine how many cells one should be analyzed to reach sufficient statistical power. Several methods have been developed using power analysis statistics, such as Scotty^1^ or web-based tools^2^, but one must estimate the number and expected frequencies of cell populations present in the sample, and such information is often not available. Therefore, these decisions are usually made based on logistical restraints (i.e., the number of cells available), financial considerations, or re-iterative experiments where an initial sample of cells is sequenced to get a sense for overall population structure, and then increasing numbers of cells are sequenced until one is satisfied that all the main populations have been identified.

## Single Nuclei Isolation and Sequencing

Single-cell RNA sequencing methods are optimal when cells can be harvested intact and viable (Grindberg et al., 2013). However, certain cell types (e.g., neurons, adipocytes), are not amenable to standard organ dissociation protocols, since enzymatic and mechanical forces easily disrupt the cytoplasmic contents (Habib et al., 2017). In these cases, an option could be to isolate intact nuclei for single-nucleus RNAseq (snRNAseq) (Grindberg et al., 2013; Habib et al., 2016, 2017; Krishnaswami et al., 2016; Lacar et al., 2016; Lake et al., 2016). To prepare single nuclei, cells are lysed with detergent and dounce homogenized to expel cytoplasmic contents and nuclei from the cellular membrane, (Habib et al., 2016), which may avoid transcriptomic changes (Van Den Brink et al., 2017). Nuclei can then be purified by flow cytometry or gradient centrifugation (Grindberg et al., 2013; Ambati et al., 2016; Habib et al., 2016). When cell-type specific nuclear proteins exist, they can be used for nuclei isolation from specific cell types using antibody labeling (Lacar et al., 2016; Habib et al., 2017).

Single-nucleus RNAseq is not only amenable for difficult to isolate cell types, but can also be used for archived tissues such as flash-frozen clinical samples. Individual nuclei isolated from frozen adult mouse and human brain tissues have been successfully sequenced, demonstrating that snRNAseq has sufficient resolution to identify many different cell types from frozen and post-mortem tissue (Grindberg et al., 2013). With the rapid development of many applications for snRNAseq, nuclei are amenable to other studies not easily done by scRNAseq.

An important question remains: To what degree is the nuclear transcriptome representative of the whole cell? Recent studies have demonstrated that many transcripts of cell and nucleus are equally represented and that nuclear RNA represents an important and significant population of transcripts that contribute greatly to the overall diversity of transcripts (Barthelson et al., 2007; Trask et al., 2009). Comparative studies of scRNAseq and snRNAseq in neural progenitor cells have also demonstrated that genes are expressed in equal proportion between whole cell and nuclei (Grindberg et al., 2013). Nanogrid single-cell and nuclei RNA sequencing studies in the same breast cancer lines found that overall copy number, expression level, and abundance had a high (*r*_s_ = 0.95) Spearman’s correlation (Gao et al., 2017). Similarly, the transcriptomes of single cells and nuclei of 3T3 cells have also demonstrated high correlation (Pearson, *r* = 0.87) (Habib et al., 2017). Together these results suggest that nuclei and cells have highly correlated relative gene expression.

Despite the similarities between single-cell and nuclei transcriptomic profiles there remain notable differences. Not surprisingly, nuclear transcriptomes are enriched for several types of nuclear RNAs (Grindberg et al., 2013; Habib et al., 2016, 2017; Krishnaswami et al., 2016; Gao et al., 2017). Since ncRNAs are only polyadenylated in the nucleus, snRNAseq provides a feasible strategy to capture the heterogeneity of ncRNA transcription in single-cell resolution (Krishnaswami et al., 2016). In addition, nuclear transcriptomes are enriched for lncRNAs and nuclear-function genes (Gao et al., 2017). Another difference between cell and nuclear RNAseq is the higher abundance of intronic sequences in snRNAseq, which ranged between 10–40% of mapped reads (Grindberg et al., 2013; Gao et al., 2017; Habib et al., 2017). These features need to be accounted for when comparing datasets from cellular versus nuclear transcriptome analyses.

In conclusion, snRNAseq has emerged as a promising avenue for profiling archived samples or cell types that are hard to viably isolate from tissues.

## Single-Cell Library Sequencing

The next critical part of designing single-cell workflows is to align the analysis pipeline with the respective NGS platform and sequencing depth. It is important to confirm that the chemistry used for library construction is compatible with the sequencing technology. Currently, there are two major outputs for libraries from scRNAseq: full-length transcript or 3′-end counted libraries, which each require different read depths (Haque et al., 2017). Full-length transcript libraries are typically sequenced at a depth of 10^6^ reads per cell, but may still yield important biological information at as low as 5 × 10^4^ reads per cell (Pollen et al., 2014). For specific applications such as alternative splicing analysis on the single-cell level, much higher sequencing depth up to 15– 25 × 10^6^ reads per cell is necessary. On the other hand, 3′-end counting libraries are sequenced at much lower depth of around 10^4^ or 10^5^ reads per cells (Haque et al., 2017). Reaching the optimal sequencing depth can be an iterative process and may require multiple rounds of optimization. Sequencing saturation can be estimated by plotting down-sampled sequencing depth in mean reads per cell (e.g., 10× Genomics Cell Ranger).

## Study Design and Data Analysis

In the following section, we highlight several key considerations from a data analysis perspective for adequately designing a successful scRNAseq study. As mentioned, many single-cell technologies can be greatly affected by technical variation, and without proper study design the results can be difficult to interpret. One critical aspect of this is the separation of *batch* and *condition*. *Batch* refers to a library that was singularly generated in a contained workflow (i.e., harvesting tissue specimen, disassociating into single-cell suspension, and generating scRNAseq library). *Condition* refers to a biological state or experimental treatment that is being analyzed in the study. Technical variation can be difficult to separate from relevant biological variation when conditions are interrogated individually. To help correct for this, the generation of replicates (biological or technical) whenever possible is strongly recommended.

In addition to replicates, an option is to mix samples and conditions within a batch, such that they can be treated without confounding each other (Hicks et al., 2015). One example is the Demuxlet workflow, where samples from genetically distinct individuals can be processed within the same library generation protocol and sequenced together (Kang et al., 2018). Prior to library generation, genotyping of distinct samples is performed and subsequently used in conjunction with the scRNAseq library to demultiplex the mixed cell sample into the samples of origin. In situations where genetically identical samples are used, or genotypic data is not readily available, cellular hashing can be employed (Stoeckius et al., 2017). This involves oligo-tagged antibodies specific to each sample in the study and then pooling and generating the scRNAseq library from the sample mixture. The antibodies labeled with unique barcodes can be traced back to its sample of origin (Stoeckius et al., 2017).

**FIGURE 2:**
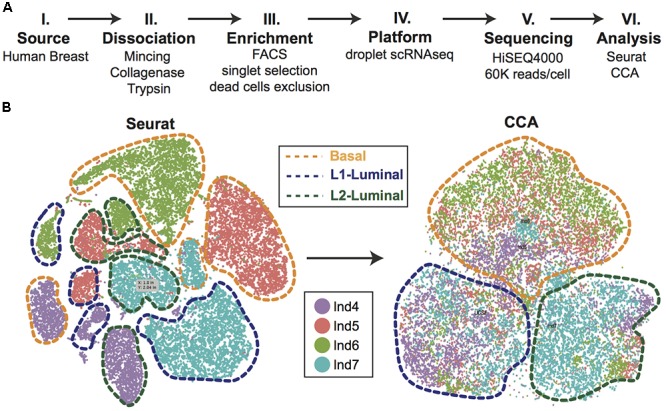
Single-cell analysis of primary human breast epithelial cells. **(A)** Approach overview summarizing individual steps of single-cell analysis approach from primary human breast epithelial cells using scRNAseq. **(B)** Combined computational analysis of 24,465 single-cell transcriptomes from primary breast epithelial cells harvested from four human individuals. Standard Seurat analysis shows clear separation by individual as shown in tSNE plot. Applying canonical correlation analysis (CCA) successfully removes individual-specific clustering giving rise to three major clusters corresponding to the three main breast epithelial cell types, namely Basal, L1-Luminal, and L2-Luminal (outlined by dotted lines).

Efforts can be made computationally to mitigate batch-to-batch variation. Batch effects are not unique to scRNAseq data, but the assumptions made by correction algorithms are not always appropriate for the bimodality of gene expression in zero-inflated scRNAseq data. Here, we highlight recent analytical frameworks that may be used to correct for this phenomenon. A recently developed approach by Haghverdi et al. (2018) builds a mixed nearest neighbor model for cells between datasets or samples that does not require known or equal proportions of cell types between data sets. In addition, the widely used Seurat pipeline for scRNAseq analysis recently employed canonical correlation analysis (CCA) that allows for discovery of co-correlated gene modules between datasets that can then be used to cluster upon (Butler et al., 2018). This approach identifies the cell types common between datasets and samples, as well as those that are unique to an individual set by finding common sources of variation in gene expression. As an illustration of this method, we applied CCA to our recently published droplet-enabled scRNAseq dataset from four individual primary human breast tissue samples (**Figure 2**). Finally, the single-cell batch correction framework MAST (Finak et al., 2015) models the positive expression mean and the over-the-background expression of transcripts, and calculates a fraction of detected genes per cell and uses this as a covariate that is independent of a previously specified control set of genes. Together, these methods serve as recent examples to handle batch-to-batch variation computationally, resulting in improved dimensionality reduction and clustering for meaningful scRNAseq data analysis.

Beyond accounting for technical variation, a common question that researchers address is the relatedness of described cell populations through the lens of a differentiation processes. The key assumption of pipelines that seek to address this is that the tissue sample analyzed using scRNAseq contains cell types/states that represent not only the ends of a differentiation process, but also stem/progenitor cells and transitional cell states along the path of differentiation. Common analysis suites that seek to reconstruct these differentiation trajectories are Monocle (Qiu et al., 2017), TSCAN (Ji and Ji, 2016), and CellTree (duVerle et al., 2016). Each use different methods, but their goal is to visualize differentiation trajectories and identify expression signatures that change through pseudotime.

## Conclusion

To fully harness the potential of single-cell analysis tools to decipher complex biological systems on the level of individual cells, careful study design and rigorous optimization of every step along the experimental procedure are mandatory. Here, we delineate a step-wise experimental approach for optimizing tissue handling, cell dissociation and enrichment, single-cell platform selection, library sequencing, and data analysis for designing single-cell workflows. A move toward standardized and automated processing of tissues will minimize changes introduced by tissue handling that may obscure biologically relevant transcriptomic profiles. For tissues that are problematic to dissociate into high-quality and viable single-cell suspensions, snRNAseq offers a solution to this problem, and can be used to achieve uniform extraction and sequencing of multiple cell types for cross comparison. Numerous computational frameworks are currently emerging that help mitigate batch effects to separate biological variation from unwanted technical variation.

## Author Contributions

KK outlined concept and overview of review. QN, NP, and KN wrote the manuscript. KK and QN designed and prepared the figures.

## Conflict of Interest Statement

The authors declare that the research was conducted in the absence of any commercial or financial relationships that could be construed as a potential conflict of interest.

## Footnotes

1 http://scotty.genetics.utah.edu/

2 http://satijalab.org/howmanycells
